# Evaluation of case definitions for estimation of respiratory syncytial virus associated hospitalizations among children in a rural community of northern India

**DOI:** 10.7189/jogh.05.020419

**Published:** 2015-12

**Authors:** Siddhartha Saha, Bharti Gaur Pandey, Avinash Choudekar, Anand Krishnan, Susan I. Gerber, Sanjay K. Rai, Pratibha Singh, Mandeep Chadha, Renu B. Lal, Shobha Broor

**Affiliations:** 1 Center for Disease Control and Prevention, Influenza Programme, New Delhi, India; 2 Manav Rachna International University, Faridabad, India; 3 AIIMS–INCLEN collaborative Influenza project, New Delhi, India; 4 Centre for Community Medicine, All India Institute of Medical Sciences, New Delhi, India; 5 Respiratory Pathogen Branch, National Center for Immunization and Respiratory Diseases, United States Centers for Disease Control and Prevention, Atlanta, Georgia, USA; 6 National Institute of Virology, Pune, India; * Equal contribution of authors

## Abstract

**Background:**

The burden estimation studies for respiratory syncytial virus (RSV) have been based on varied case definitions, including case–definitions designed for influenza surveillance systems. We used all medical admissions among children aged 0–59 months to study the effect of case definitions on estimation of RSV–associated hospitalizations rates.

**Methods:**

The hospital–based daily surveillance enrolled children aged 0–59 months admitted with acute medical conditions from July 2009–December 2012, from a well–defined rural population in Ballabgarh in northern India. All study participants were examined and nasal and throat swabs taken for testing by real–time polymerase chain reaction (RT–PCR) for RSV and influenza virus. Clinical data were used to retrospectively evaluate World Health Organization (WHO) case definitions (2011) commonly used for surveillance of respiratory pathogens, ie, acute respiratory illness (WHO–ARI), severe ARI (SARI) and influenza–like illness (ILI), for determination of RSV–associated hospitalization. RSV–associated hospitalization rates adjusted for admissions at non–study hospitals were calculated.

**Findings:**

Out of 505 children enrolled, 82 (16.2%) tested positive for RSV. Annual incidence rates of RSV–associated hospitalization per 1000 children were highest among infants aged 0–5 months (15.2; 95% confidence interval (CI) 8.3–26.8), followed by ages 6–23 months (5.3, 95% CI 3.2–8.7) and lowest among children 24–59 months (0.5, 95% CI 0.1–1.5). The RSV positive children were more likely to have signs of respiratory distress like wheeze, chest in–drawing, tachypnea, and crepitation compared to RSV–negative based on bivariate comparisons. Other less commonly seen signs of respiratory distress, ie, nasal flaring, grunting, accessory muscle usage were also significantly associated with being RSV positive. Compared to the estimated RSV hospitalization rate based on all medical hospitalizations, the WHO–ARI case definition captured 86% of the total incidence, while case definitions requiring fever like ILI and SARI underestimated the incidence by 50–80%.

**Conclusions:**

Our study suggests that RSV is a substantial cause of hospitalization among children aged <24months especially those aged <6 months. The WHO–ARI case definition appeared to be the most suitable screening definition for RSV surveillance because of its high sensitivity.

Globally, respiratory syncytial virus (RSV) is a leading cause of acute lower respiratory infection (ALRI) including bronchiolitis and pneumonia among young children [1,2]. Studies indicate that most children are infected by RSV in the first two years of life with infants bearing the highest rates of RSV–associated lower respiratory illness [3,4]. A recent meta–analysis estimated 3.4 million hospitalizations and 66 000–199 000 RSV–associated deaths among children <5 years of age with ALRI, with 99% of the deaths occurring in developing countries [5]. While there are some studies on burden of pneumonia and viral etiology in India and other developing countries [2,6], recent data from a community–based and hospital–based studies have further emphasized the importance of RSV among children <5 years of age [7-9].

Systematic data are needed to better understand seasonality, burden and mortality associated with RSV infection in children. RSV infection has a different clinical presentation, age distribution, risk factors, and seasonality compared to influenza infection and requires studies specifically designed to detect and evaluate RSV [3,4]. For example, RSV illness may present without fever particularly among infants, whereas influenza is more likely to present as a febrile illness and thus, fever may be included in the case definitions [10]. However, existing surveillance networks for influenza, with protocols and case definitions designed for influenza have also often been used to generate burden estimations for RSV [11]. Thus, there is a need to identify appropriate case–definitions for epidemiologic field studies to accurately estimate the RSV burden among children.

The presence of a broad platform to estimate the rates of hospitalized influenza which captured all–cause hospitalization in a well–defined population with health demographic surveillance system [12] enabled us to evaluate the sensitivity and specificity of different case definitions and RSV burden among children <5 years in a rural setting in northern India. The current paper utilizes data from this hospital–based surveillance study to evaluate case definitions for RSV detection and the impact of choice of case definitions on RSV hospitalization rate estimates among children aged <5 years in a rural setting in India.

## METHODS

### Study site

The Ballabgarh Health and Demographic Surveillance System (HDSS) site is about 40 km south of Delhi and comprised a population of about 90 000 in June 2011 including 9500 children 0–59 months of age in 28 villages [13]. Based on health utilization survey of the site population conducted in April 2009, three public hospitals and 30 private facilities (ranging in size from 5–35 beds) in Ballabgarh and Faridabad towns were included for daily surveillance for patients from the catchment area seeking inpatient care [12,14]. Immunization coverage for EPI vaccines (BCG, DPT, OPV, Hepatitis B and Measles) provided through public health facilities was >95% in the study villages [13]; Hib vaccine was introduced into public health program in 2012–2013. Coverage for pneumococcal and rota vaccines are not known but likely to be low as they are available only in private facilities.

### Enrolment and data collection

During July 2009 – December 2012, hospital–based daily surveillance–enrolled children aged 0–59 months from Ballabgarh–HDSS area who were hospitalized overnight with any acute medical illness or acute exacerbation of chronic illness at participating medical facilities [8,12]. Trained study physicians collected data using a standardized form on demographics, medical history and clinical symptoms by interview of the caregivers, and extracted data on clinical signs at admission from the medical record followed by clinical examination of cases for additional clinical information.

### Study definitions

The presence of fever or key respiratory signs or symptoms was determined among all hospitalized children aged 0–59 months. Fever was defined as either measured temperature >38.0°C at admission or parental report of fever because antipyretic use is known to be common in the study community [15]. Key respiratory symptoms or signs were defined as parental report of cough or fast breathing or physician exam findings of tachypnea, crepitation, wheezing, nasal flaring, chest in–drawing, grunting, or stridor. Tachypnea was defined based on the definition used by the Indian Integrated Management of Neonatal and Childhood Illness (IMNCI) as ≥60 breaths/min in children aged 0–2 months of age, ≥50 breaths/min in children aged 2–12 months of age, and ≥40 breaths/min in children aged 12 months –5 years of age [16]. Data on clinical signs and symptoms were used *post hoc* to classify each patient using standard case definitions specified by WHO (May 2011, see **Box 1**), ie, acute respiratory infection (ARI), severe acute respiratory infection (SARI), and influenza–like illness (ILI), and evaluated them for RSV positivity.

Box 1 **Case definitions for respiratory syndromes [17,18]**

**ARI** – acute respiratory infection (WHO, 2011):

Acute onset of at least one of the following four respiratory symptoms: cough or sore throat or shortness of breath or coryza and a clinician’s judgment that illness is due to infection.

**ILI** – influenza–like illness (WHO, 2011):

An acute respiratory illness with onset during the last 7 days with measured temperature ≥38°C, AND cough. (Dropped sore throat).

**ILI** (old):

Sudden onset of fever (>38°C) with cough or sore throat, in absence of other diagnoses.

**SARI** – severe ARI (WHO, 2011):

An acute respiratory illness with onset during the previous 7 days requiring overnight hospitalization that includes history of fever or measured fever of ≥38°C, AND cough, AND shortness of breath or difficulty breathing.

**SARI** (old):

Meets ILI (old) definition (sudden onset of fever (>38°C) with cough or sore throat) and has shortness of breath or difficulty breathing and requires overnight hospitalization.

**Pneumonia** (IMCI):

Fast breathing or chest indrawing

**Severe pneumonia** (IMCI):

General danger signs – Not able to drink, persistent vomiting, convulsions, lethargic or unconscious, stridor, or severe malnutrition

### Specimen collection and laboratory methods

Nasal and throat samples were collected by a study nurse from all enrolled patients within 24 hours of admission to the hospital using polyester swabs; in infants only nasal swabs were collected. The swabs were placed immediately into viral transport media and transported on ice to laboratory on the same day for processing. Specimens were tested for RSV and influenza using US Centers for Disease Control and Prevention (CDC) real–time reverse transcription polymerase chain reaction (rRT–PCR) protocols, as described previously [8,12].

### Data analysis

We assessed different signs and symptoms associated with RSV positivity using bivariate analysis for different age–groups and backward stepwise logistic regression adjusted for age–groups. We also assessed the ability of standard case definitions (ARI, SARI, ILI) for respiratory illness to capture RSV–associated hospitalizations by calculating sensitivity and specificity for each case definition using all RSV positive hospitalized patients as the gold standard. We assessed the impact of standard case definitions on average annual incidences of RSV–associated hospitalizations using available 3 calendar years’ data from 2010 to 2012.

The population of June 2011 in the HDSS was considered the mid–term population denominator for calculations. Annual health utilization surveys were used to estimate the average proportion of hospitalization in enrolled facilities [14]. The annual hospitalization rates based on enrollment were adjusted for missed hospitalizations in non–study facilities and were multiplied by the positivity rate of the viruses to get an estimate of virus specific hospitalization rates. We calculated incidence rates for four age groups (0–5 months, 6–11 months, 12–23 months and 24–59 months) for RSV and influenza because clinical manifestations, as well as viral etiologies are likely to be different in these age groups. The average annual incidence was calculated separately for each case definition among all medical and respiratory admissions, to evaluate the effect of using different screening definitions on the estimations of RSV–associated hospitalizations. The data analysis was done using STATA 12 (College Station, Texas, USA) [17] and Micosoft Excel. A *P*–value of <0 · 05 was considered statistically significant for all analyses. The 95% confidence intervals (CI) for odds ratios, sensitivity and specificity were calculated.

## RESULTS

### Background characteristics

During the study period, 505 children aged 0–59 months from the HDSS area hospitalized with acute medical illness in the health facilities under surveillance were enrolled; of these 79.6% (402/505) were aged 0–24 months and 71.7% (362/505) were males (**Table 1**). RSV was detected in 82 (16%) hospitalized patients with 89% (73/82) of detections among children <2 years old (*P* < 0.001). There was no significant difference in gender, time from symptom onset to specimen collection or any underlying medical condition among RSV negative and positive children (with the exception of chronic diarrhea observed among RSV negative children, data not shown). The RSV detections among hospitalized children occurred with seasonal peaks between September–October and then again in January–February of each year (data not shown).

**Table 1:** Characteristics of children <5 years of age enrolled for all cause hospitalization in Ballabgarh, Haryana, India from July 2009 – December 2012

Characteristics (n, %)	Total (n = 505)	RSV+ (n = 82)	RSV– (n = 423)	*P–*value
**Age group (months):**				
0–5	114 (22.6)	35 (42.7)	79 (18.7)	<0.001
6–11	146 (28.9)	16 (19.5)	130 (30.7)	
12–23	142 (28.1)	22 (26.8)	120 (28.4)	
24–35	43 (8.5)	4 (4.9)	39 (9.2)	
36–59	60 (11.9)	5 (6.1)	55 (13)	
**Sex:**				
Female	143 (28.3)	24 (29.3)	119 (28.1)	0.834
Male	362 (71.7)	58 (70.7)	304 (71.9)	
Time from symptom onset to specimen collection:*				
0–2 days	127/468 (27.1)	16/79 (20.2)	111/389 (28.5)	0.497
3–4 days	135/468 (28.8)	26/79 (32.9)	109/389 (28.0)	
5–7 days	116/468 (24.8)	21/79 (26.6)	95/389 (24.4)	
8–10 days	39/468 (8.3)	9/79 (11.4)	30/389 (7.7)	
≥11 days	51/468 (10.9)	7/79 (8.9)	44/389 (11.3)	

*Data on 37 cases, including 3 RSV–positive cases, were missing.

### Clinical characteristics

Among the enrolled children, 347 (68.7%) had some respiratory illness. Further, those with symptoms of cough (OR = 5.3, 95% CI 2.8–10.1) and fast breathing (OR = 3.9, 95% CI 2.4–6.3) were more likely to test positive than negative for RSV (**Table 2**). The presence of signs of wheeze, chest in–drawing, tachypnea, and crepitation had significantly higher odds of being RSV positive vs RSV–negative based on bivariate comparisons. Other less commonly seen signs of respiratory distress, ie, nasal flaring, grunting, accessory muscle usage were also significantly associated with being RSV positive. Stepwise backward logistic regression (**Table 3**) analysis of all clinical features identified the presence of cough, fast–breathing, crepitation and hypoxia as significant predictors for RSV–associated hospitalization, while history of fever and diarrhea were significantly associated with non–RSV–associated hospitalization.

**Table 2:** Age–specific clinical signs and symptoms significantly associated with laboratory–confirmed respiratory syncytial virus infection among hospitalized patients <5 y of age, in Ballabgarh, India, July 2009 – December 2012; (n = 505)*

	0–5 months		6–23 months		24–59 months		0–59 months	
	**RSV+ (n = 35)**	**OR (95% CI)**	**RSV+ (n = 38)**	**OR (95% CI)**	**RSV+ (n = 9)**	**OR (95% CI)**	**RSV+ (n = 82)**	**OR (95% CI)**
**Symptoms:**								
Fever	27 (77.1)	0.6 (0.2–1.6)	32 (84.2)	0.8 (0.3–2.2)	9 (100.0)	–	68 (82.9)	0.8 (0.4–1.5)
Cough	32 (91.4)	4.4 (1.2–15.7)	32 (84.2)	6.5 (2.6–16.0)	6 (66.7)	1.6 (0.4–6.8)	70 (85.4)	5.3 (2.8–10.1)
Breathing difficulty	20 (57.1)	3.5 (1.5–7.9)	15 (39.5)	3.3 (1.6–6.9)	2 (22.2)	1.5 (0.3–8)	37 (45.1)	1.8 (1.1–2.7)
Nasal discharge	13 (37.1)	1.4 (0.6–3.1)	20 (52.6)	2.3 (1.1–4.5)	4 (44.4)	2 (0.5–8)	37 (45.1)	1.8 (1.1–2.9)
Sore throat (>2years)	0 (0)	–	0 (0)	–	2 (22.2)	1.5 (0.3–8)	2 (16.7)	1.4 (0.3–7.1)
Ear discharge	1 (2.9)	1.1 (0.1–12.9)	4 (10.5)	2.3 (0.7–7.6)	0 (0)	–	5 (6.1)	1 (0.4–2.7)
Fast breathing	18 (51.4)	2.9 (1.3–6.7)	18 (47.4)	6.1 (2.9–12.8)	3 (33.3)	3.8 (0.8–17.3)	39 (47.6)	3.9 (2.4–6.3)
Lethargy	9 (25.7)	1.5 (0.6–3.9)	3 (7.9)	0.3 (0.1–0.9)	3 (33.3)	1.2 (0.3–5)	15 (18.3)	0.7 (0.4–1.2)
Refusal to feed	15 (42.9)	1 (0.5–2.3)	10 (26.3)	0.6 (0.3–1.3)	3 (33.3)	0.8 (0.2–3.3)	28 (34.2)	0.8 (0.5–1.3)
Seizure	2 (5.7)	0.9 (0.2–4.9)	0 (0)	–	2 (22.2)	6.4 (1–41.4)	4 (4.9)	1.6 (0.5–4.7)
Unconsciousness	2 (5.7)	0 (0–0)	0 (0)	–	0 (0)	–	2 (2.4)	1.5 (0.2–11)
Vomiting	10 (28.6)	0.4 (0.2–0.9)	22 (57.9)	0.4 (0.2–0.8)	4 (44.4)	0.5 (0.1–2)	36 (43.9)	0.4 (0.2–0.6)
Diarrhea	13 (37.1)	0.5 (0.2–1.2)	16 (42.1)	0.2 (0.1–0.4)	1 (11.1)	0.2 (0–1.7)	30 (36.6)	0.3 (0.2–0.5)
Rash	1 (2.9)	2.3 (0.1–37.8)	0 (0)	–	0 (0)	–	1 (1.2)	0.3 (0–2.6)
Jaundice	1 (2.9)	1.1 (0.1–12.9)	0 (0)	–	0 (0)	–	1 (1.2)	0.7 (0.1–4.2)
**Signs:**								
Fever	1 (2.9)	0.2 (0–1.3)	10 (26.3)	1.8 (0.8–4)	6 (66.7)	5 (1.2–21.3)	17 (20.7)	1.1 (0.6–2)
Stridor	4 (11.4)	2.4 (0.6–10.3)	5 (13.2)	9.3 (2.4–36.5)	0 (0)	0 (0–0)	9 (11)	5.7 (2.2–14.8)
Nasal flaring	5 (14.3)	2.4 (0.6–8.9)	4 (10.5)	9.7 (2.1–45.2)	0 (0)	0 (0–0)	9 (11)	5.1 (2–12.9)
Chest in–drawing	13 (37.1)	5.1 (1.2–13.9)	6 (15.8)	3.7 (1.3–10.6)	0 (0)	0 (0–0)	19 (23.2)	5.7 (2.9–11.3)
Grunting	5 (14.3)	3 (0.8–12.1)	4 (10.5)	7.2 (1.7–30.3)	0 (0)	0 (0–0)	9 (11)	5.6 (2.2–14.7)
Accessory muscle use	5 (14.3)	4.2 (0.9–18.8)	1 (2.6)	0.8 (0.1–6.7)	0 (0)	0 (0–0)	6 (7.3)	2.5 (0.9–6.8)
Crepitation	21 (60)	2.9 (1.3–6.6)	17 (44.7)	5.0 (2.4–10.3)	4 (44.4)	5.5 (1.3–23.2)	42 (51.2)	5 (3–8.2)
Wheeze	24 (68.6)	5 (2.1–11.8)	10 (26.3)	2.8 (1.2–6.4)	2 (22.2)	2.2 (0.4–11.7)	36 (43.9)	4.5 (2.7–7.5)
Tachypnoea	25 (71.0)	1.4 (0.6–3.3)	16 (42.1)	1.9 (0.9–3.8)	1 (11.1)	0.6 (0.1–4.8)	42 (51.2)	2.2 (1.4–3.5)
Hypoxia*	8 (22.9)	1.5 (0.6–4)	8 (22.9)	2.6 (1.0–6.6)	1 (11.1)	2.8 (0.3–28.3)	17 (20.7)	2.5 (1.3–4.8)

OR – odds ratio, CI – confidence interval

*Defined as oxygen saturation <90% on room air or <95% on oxygen therapy.

**Table 3:** Clinical predictors of respiratory syncytial virus associated hospitalization among children aged <5 y by stepwise backward logistic regression adjusted for age–groups*

Symptoms and signs	Odds ratio (95% CI)	*P* > z
Cough	1.85 (1.13–0.46)	0.013
History of fever	0.4 (0.23–0.11)	0.001
History of fast breathing	3.44 (2.26–0.73)	<0.001
Diarrhea	0.62 (0.41–0.12)	0.021
Inability/refusal to feed	0.57 (0.39–0.1)	0.004
Unconsciousness	0.07 (0.01–0.07)	0.005
Nasal flaring	3.13 (1.41–1.27)	0.005
Stridor	2.73 (1.22–1.12)	0.014
Accessory muscle use	0.22 (0.08–0.11)	0.003
Crepitation	2.79 (1.81–0.61)	<0.001
Hypoxia	2.71 (1.58–0.74)	<0.001

CI – confidence interval

*Age groups: 0–5 months, 6–11 months, 12–23 months and 24–59 months.

### Case definitions

**Figure 1:**
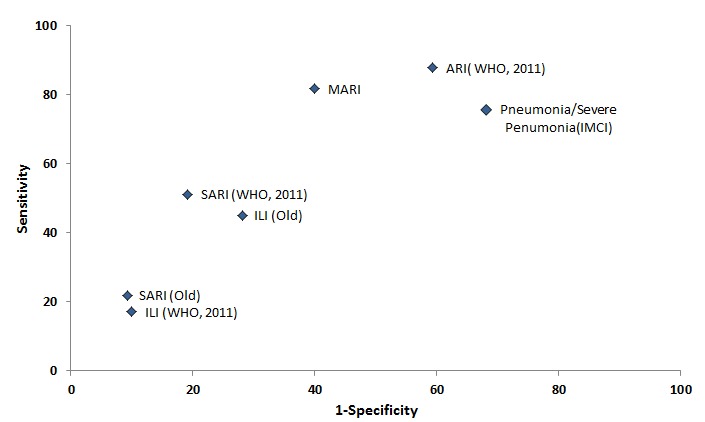
Receiver operator characteristic (ROC) chart for screening definitions for respiratory syncytial virus associated hospitalization with 1–specificity on x-axis and sensitivity on y-axis. Each dot represents the sensitivity and 1–specificity of the case definition for detection of RSV–associated hospitalization. See **Box 1** for case definitions details of each syndrome.

We then examined the sensitivity and specificity of standard case definitions (ARI, SARI, ILI) for detection of RSV–associated hospitalization (**Figure 1**). Among the standard case definitions, ARI had the highest sensitivity (87.8%) and specificity (40%) based on receiver–operating characteristics. All other case definitions (ILI, and SARI, (both old and revised 2011 versions) had lower sensitivity and variable specificity, with older the definition of SARI showing higher specificity). We also evaluated different clinical syndromes including IMCI definitions of pneumonia/severe pneumonia [18] and other syndromes. The sensitivity of IMCI either pneumonia or severe pneumonia was high (75.6%) but specificity was low (31.9%). Among other combination of symptoms and signs, we found history of cough or crepitation along with presence of any one of following– history of fast–breathing, breathing difficulty, nasal discharge, sore–throat, chest–in–drawing, wheeze or hypoxia, had high sensitivity (81 · 7%) and specificity (60%).

### Burden of RSV in children

**Figure 2:**
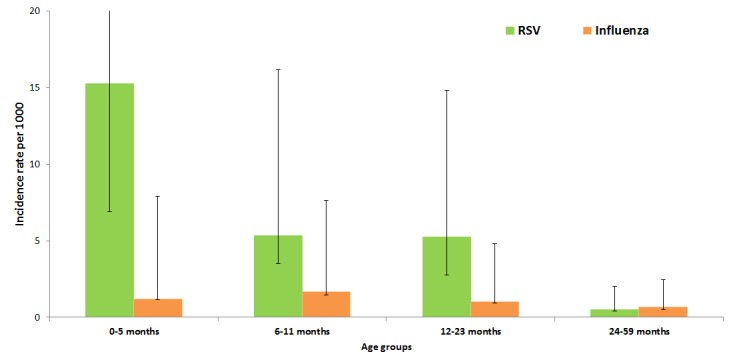
Age–specific incidence rates using all–cause hospitalization for respiratory syncytial virus (RSV) and influenza–associated hospitalization rates in north India (2009–2012). The age groups are on the x–axis and incidence rate of RSV–associated hospitalization per 1000 children denoted on the y–axis. Green and orange colored bars denote incidence rates of RSV and influenza associated hospitalizations respectively with error bars for 95% confidence intervals.

The average annual incidence of RSV–associated hospitalizations was found to be higher at 7 · 4 (95% CI: 4.9–10 · 5) per 1000 child–years in those 0–23 months as compared to 0.5 (95% CI 0.1–1.5) among 24–59 months population signifying that most of burden of RSV–associated hospitalization is among children under two years of age. Further breakdown of the age–specific annual incidence of RSV–associated hospitalization per 1000 children revealed that the highest rate occurred in young infants 0–5 months (OR = 15.2, 95% CI 8.3–26.8), followed by 6–23 months (OR = 5.3, 95% CI 3.2–8.7) with comparable rates for the 6–11 months and 12–23 months age groups (**Figure 2**). Incidence rates for influenza were lower across all age groups.

### Impact of case definitions on RSV burden

We assessed the impact of the use of different standard case definitions on RSV burden estimates by comparing the definitions with RSV hospitalization based on all–cause hospitalization (**Table 4**). Use of the ARI case definition among hospitalized children would have detected 90% and 86% of the RSV–associated hospitalization rates in children aged <2 years and <5 years, respectively. In contrast, use of definitions which require presence of fever and most commonly used for influenza surveillance platforms, ie, SARI or ILI definitions, (both old and revised) would have under estimated RSV burden by as much as 50–85% in both <2 as well as <5–year age groups (**Table 4**).

**Table 4:** Effect of screening case definition on respiratory syncytial virus (RSV) associated annual hospitalization rates (2010–2012)*

Population under surveillance	Under–2 years (N = 3956)				Under–5 years (N = 9740)			
**Case definitions**	**No. met case definition**	**No. of RSV positive cases (%)**	**Incidence Rate (IR)†**	**IR under–estimation (%)**	**No. met case definition**	**No. of RSV positive cases (%)**	**IR†**	**Under–estimation IR (%)**
All Medical	386	67 (17)	7.4	NA	484	74 (15)	3.2	NA
ARI (WHO)	239	60 (25)	6.6	–10%	299	64 (21)	2.8	–14%
SARI (WHO)	90	34 (38)	3.8	–49%	107	35 (33)	1.5	–53%
SARI (Old)	35	10 (29)	1.1	–85%	41	11 (27)	0.5	–85%
ILI (WHO)	37	10 (27)	1.1	–85%	54	14 (26)	0.6	–81%
ILI (Old)	106	26 (25)	2.9	–61%	139	32 (23)	1.4	–57%

ARI – acute respiratory illness, SARI – severe acute respiratory illness, ILI – influenza–like illness, WHO – World Health Organization

*For incidence rate calculations data for full calendar years were used. Data imputed for 6 weeks surveillance gap between 31 January 2012 and 13 March 2012.

†Per 1000 age–specific population.

## DISCUSSION

The uniqueness of this study based on a comprehensive surveillance system designed to capture all–cause hospitalization in a well–defined population, together with the availability of highly sensitive molecular testing for RSV allowed us to estimate RSV–associated the hospitalization rates among children aged <5 years in rural northern India [8,12]. Most studies estimating RSV–associated burden rely on existing surveillance platforms for influenza in developing countries [11,19]. A very important aspect of our study was that we were able to evaluate the impact of using different case definitions on burden estimation. We provided evidence that the WHO–defined ARI case definition has the highest sensitivity for RSV–associated hospitalization and demonstrate the limitations of definitions like ILI and SARI commonly used for influenza surveillance. We found that testing only children meeting the SARI and ILI definitions would have under–estimated the burden of RSV–associated hospitalization by almost 50–85%, although specificity would have been significantly higher with the latter case definition. Several case definitions, including ARI, have been used in studies for RSV burden estimation in many countries [11,19-22]. This highlights the importance of the use of a sensitive case definition for surveillance of RSV to avoid underestimation of the burden.

The all–cause hospitalization surveillance also allowed us to compare the symptoms and signs of hospitalized children with or without RSV and thereby identify the clinical predictors for RSV–associated hospitalization in children; this would not have been possible if only standard case definitions were used to identify potential RSV patients. We found that the presence of cough, fast–breathing, crepitation and hypoxia are independent predictors of RSV infection. Of note, Durani et al. (2008) in their study among children hospitalized with ARI found the combination of cough, wheezing and retractions to be good clinical predictor for RSV infection [23]. We found that even though fever is a common presenting symptom and sign among children being hospitalized, it is not a good predictor of RSV–associated hospitalization in this population. The use of history or presence of fever in screening case definitions lowers the sensitivity of the definition. We also observed that two–thirds of hospitalized patients had some respiratory symptoms, suggesting that a very high proportion of hospitalizations are due to respiratory symptoms in rural India. This observation corroborates previous findings that ARI is a significant cause of morbidity in the developing world [4,24,25].

We found substantial incidence of RSV–associated hospitalization in the study community especially among <2–year old children. The RSV–associated incidence of hospitalization per 1000 child–years was 3.2 among <5–year children, and 7.4 among <2–year children, with highest incidence rate of 15.2 per 1000 child years among infants 0–5 months, which is similar to findings of an earlier community–based study from this area [7]. RSV–associated hospitalization rates among children <5years observed in our study were also comparable to what has been observed in Kenya (2.9/1000 child–years) and Guatemala (2–13.7/1000 child–years), although lower than in Thailand (9.8/1000 child–years), Indonesia (34/1000 child–years), Nigeria (94/1000 child–years) [20-22,26]. The highest risk group for RSV–associated burden was infants 0– to 5–month old, which was also observed in Thailand (15.4/1000 child–years), Indonesia (41/1000 child–years), Hong Kong (<6m, 23.4–31.1/1000 child–years), Guatemala (5.9–45.9/1000 child–years), Kenya (11.0/1000 child–years) and Nigeria (116/1000 child–years). Studies from Brazil, USA and Korea have established that infants are at high risk of RSV–associated burden both in terms of incidence in community and proportion of hospitalization [19-22,27-31]. Even though the rates of RSV–associated hospitalization vary in different countries, most of the burden is observed among children aged <2 years, therefore studies focusing on RSV–associated morbidity and mortality or high–risk group for RSV infections may consider children aged <2 years. Also, prioritizing this age–group for any preventive measure would likely have profound effect on prevention of RSV–associated hospitalization and deaths in India and other developing countries [32,33].

The study’s limitations include first that it was designed to address influenza–associated burden, so data on variables such as gestation at birth were not collected; this data might have allowed us to also understand some of the risk–factors for RSV infection. Second, the active surveillance at health facilities (which were almost 30 plus facilities) did not capture all hospitalizations for the denominator population, and we had to make adjustments in rates of hospitalization using HDSS survey results. It is plausible that children seeking care at participating hospitals may be different from those who did not seek care in these hospitals, thus biasing the incidence rates for RSV. Third, there is a possibility that we have under–estimated the burden of RSV–associated hospitalization as some children might not have been hospitalized in spite of being diagnosed with severe respiratory illness [11,24]. Despite these limitations, we believe that this study enabled us to understand the effect of surveillance case definitions on population–based rates of RSV hospitalization in northern rural India. However, due to the study design where we captured all cause medical hospitalization instead of ILI or SARI, we are in unique position to not only address the burden of RSV, but also assess various screening case definition for RSV–associated hospitalization. Analysis of this broad platform for RSV case definition assessment allowed us to recommend the WHO–defined ARI case definition as the most appropriate screening case definition for RSV among those considered.

## CONCLUSION

In conclusion, we observed that RSV is a substantial cause of hospitalization among children aged <2 years, and especially among infants aged <6 months. This is true regardless of the screening definition used, even though rates may be underestimated if an insensitive screening definition is used. These data will help support public health strategies and interventions, targeting young children to reduce the overall RSV–associated morbidity and mortality among children in the developing world.
